# High-resolution mapping of CH_4_/N_2_O emissions from industrialization-related anthropogenic sources in China

**DOI:** 10.1093/nsr/nwae481

**Published:** 2025-01-06

**Authors:** Ziyang Lou, Haoyu Zhang, Xu Zhao, Qiang Liu, Minqi Liang, Hui Wang, Huiwen Yang, Bofeng Cai, Jingyi Lu, Ying Cui, Jingyi Wu, Fei Teng, Xiao Lu, Wenping Yuan, Mengyao Liu

**Affiliations:** Shanghai Engineering Research Center of Solid Waste Treatment and Resource Recovery, School of Environmental Science & Engineering, Shanghai Jiao Tong University, China; China-UK Low Carbon College, Shanghai Jiao Tong University, China; China Institute for Urban Governance, Shanghai Jiao Tong University, China; Shanghai Engineering Research Center of Solid Waste Treatment and Resource Recovery, School of Environmental Science & Engineering, Shanghai Jiao Tong University, China; Institute of Blue and Green Development, Shandong University, China; Institute of Energy, Environment and Economy, Tsinghua University, China; School of Atmospheric Sciences, Guangdong Province Data Center of Terrestrial and Marine Ecosystems Carbon Cycle, Sun Yat-sen University, China; Shanghai Engineering Research Center of Solid Waste Treatment and Resource Recovery, School of Environmental Science & Engineering, Shanghai Jiao Tong University, China; Institute of Blue and Green Development, Shandong University, China; Center for Climate Change and Environmental Policy, Chinese Academy for Environmental Planning, China; Shanghai Engineering Research Center of Solid Waste Treatment and Resource Recovery, School of Environmental Science & Engineering, Shanghai Jiao Tong University, China; School of College of Environmental and Chemical Engineering, Shanghai University of Electric Power, China; China-UK Low Carbon College, Shanghai Jiao Tong University, China; Institute of Energy, Environment and Economy, Tsinghua University, China; School of Atmospheric Sciences, Guangdong Province Data Center of Terrestrial and Marine Ecosystems Carbon Cycle, Sun Yat-sen University, China; Institute of Carbon Neutrality, Sino-French Institute for Earth System Science, College of Urban and Environmental Sciences, Peking University, China; Satellite Observation Department, Royal Netherlands Meteorological Institute, the Netherlands

CH_4_ and N_2_O are critical non-CO_2_ greenhouse gases (GHGs) that collectively contribute to more than one-quarter of anthropogenic global warming, accounting for ∼35%–45% of the total climate forcing [1]. China is the world's largest emitter of CH_4_ and N_2_O, accounting for 17.88% and 16.67% of global emissions, respectively [2]. As the largest developing and industrial country, China has the most comprehensive industrial supply chain globally, resulting in huge energy consumption, diverse industries, and amounts of waste and wastewater generated [3]. To achieve carbon neutrality and net-zero pledges targets outlined in the United Nations Framework Convention on Climate Change, it is crucial to adopt more precise methods for investigating the CH_4_ and N_2_O emission pathways of the industrialization-related sectors, i.e. energy, industrial processes and product use (IPPU), and the waste sector. The combined consideration of these sectors might provide a prioritized GHG mitigation strategy to respond to national ambitious targets. Elucidating the relationships among these sectors and their spatial/temporal distributions from a bottom-up perspective could clarify the critical point of emission processes and potential control measurements, and provide the basic information to balance economic development with industrial adjustment processes. In this study, we provided a more detailed, point-source level bottom-up estimation of CH_4_ and N_2_O emissions from industrialization-related anthropogenic sources from 2000 to 2022 at a 0.1° × 0.1° spatial resolution. Our approach offered a more continuous timeline and spatial coverage based on a comprehensive database and localized emission factors (the detailed methodologies are provided in the Supplementary Data online and Tables S1, S2). The contributions of these sectors to CH_4_ and N_2_O emissions were quantified, and potential mitigation policies were proposed from the perspective of GHG emissions.

The general CH_4_ and N_2_O emission inventories are shown in Fig. 1, totaling 31.26 and 1.15 Tg N_2_O yr^−1^ (equivalent to 913.44 and 313.85 Tg CO_2_-eq yr^−1^) in 2022, respectively. The energy, IPPU and waste sectors contributed around 68.25%, 11.92% and 19.83% of total emissions, respectively (Fig. S1c). For CH_4_ emissions, ∼21.18 and ∼3.64 Tg CH_4_ yr^−1^ were released from the solid fuels and oil & natural gas systems, respectively. Of these emissions, 20.06 and 2.83 Tg CH_4_ yr^−1^ came from fugitive emissions due to the mining process. Wastewater treatment plants (WWTPs), including those treating domestic and industrial wastewater, and landfills, generated around 2.73 and 3.71 Tg CH_4_ yr^−1^, as two byproducts from our daily lives and industrial production [4,5]. With regards to N_2_O emissions, 0.54 Tg N_2_O yr^−1^ were generated from IPPU processes, accounting for 46.62% of total N_2_O emissions, which were mainly from adipic acid production via the oxidation of the cyclohexanone/cyclohexanol mixture and nitric acid production through NH_3_ oxidation [6,7] (Fig. S1b). WWTPs contributed ∼22.13% of total N_2_O emissions, because of the widespread application of denitrification/nitrification processes. Both key industrial processes and WWTPs were crucial for reducing N_2_O emissions. Specifically, CH_4_ and N_2_O emissions originated from different sources and processes, necessitating separate identification for precise implementation of mitigation strategies.

**Figure 1.**
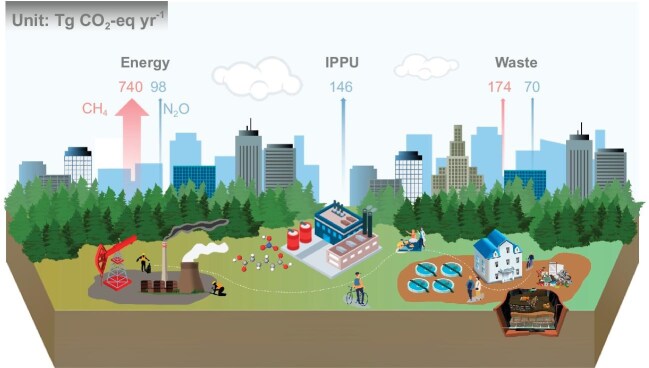
CH_4_ and N_2_O emissions from China's industrialization-related anthropogenic sources including energy, industrial processes and product use (IPPU), and waste sectors in 2022.

The gaps between our inventory and the National Greenhouse Gas Inventories (NGHGIs) could be used to identify the key factors of GHG estimation [8]. A similar pattern of CH_4_ and N_2_O emissions was observed, while the overall emissions in our results were lower, with an average value of 88.05% of those listed in the NGHGIs (Fig. S2 and Tables S3, S4). This discrepancy could be attributed to the more detailed point-source data obtained; for example, CH_4_ emissions from underground coal mines were calculated using mine-specific emission factors in this study (detailed site-specific data are shown in Supplementary Data 3.1 and 3.3). Moreover, our estimates offered a more continuous timeline and spatial coverage, with the capability for rapid updates compared with NGHGIs (see Supplementary Data).

The geographical distribution of the sum of CH_4_ and N_2_O emissions is shown in Fig. S3, revealing two major high-density regions in North and Southwest China. Spatial variation was mainly driven by the distribution of fossil energy resources (particularly coal) and industrial structure. For example, Shanxi and Guizhou, the top two emitters, accounted for 22.46% and 9.11% of China's total emissions, respectively. In these provinces, >86.60% of their emissions came from the energy sector, as they were the main coal excavated sources in China. Localized mitigation policies should be harmonized with the energy industrial strategy to support green transition. One approach is to promote the development of coal bed methane to reduce the emission intensity of coal production while shifting the energy mix to low-carbon energy. Provinces such as Shandong, Chongqing and Henan, each contributing ∼5.45% to the country's total emissions, were primarily driven by IPPU. For these regions, implementing end treatment technologies such as the thermal decomposition method could reduce emissions from industrial sources. Waste sectors in developed southeastern provinces like Zhejiang and Guangdong contributed significantly to GHGs due to the higher material consumption [9]. Mitigation processes, such as improving landfill gas collection and flaring, could contribute to a significant reduction of CH_4_ emissions in these regions. The local emissions from the energy sector in these provinces were low, with the support of national policies like the West-to-East Gas Pipeline.

The long-term trend showed a significant increase in CH_4_ emissions ranging from 11.61 to 31.26 Tg CH_4_ yr^−1^ and N_2_O emissions ranging from 0.14 to 1.15 Tg N_2_O yr^−1^ during 2000–2022 (Fig. S4). Three phases were generally observed, i.e. both increased sharply in the first decade (2000–2010) because of rapid energy consumption, and then fluctuated as a result of the balance between GHG control measures, continued increases in energy extraction and IPPU, and the rapid rise in per capita daily waste and wastewater generation. After that, the closure of numerous small-scale coal mines led to a reduction in relative GHG emissions, while N_2_O emissions from the waste sector increased greatly, as the wastewater collection and treatment rate increased by ∼83.20% over the past decade, reaching a total of 0.22 billion tons per day in 2022 [9].

Future work on CH_4_/N_2_O emissions estimation and mitigation should focus on capturing more details of emission sources and taking targeted mitigation measures. For instance, emission factors in the wastewater sector should be adjusted to incorporate local operational practices, management levels and climate conditions. Given the heterogeneity and substantial variations in CH_4_ and N_2_O emissions over the past decades, implementing long-term and real-time monitoring at typical sites (such as coal mines, chemical production plants and landfills) is essential for accurate data collection and analysis for specific locations [10]. ‘GHG control and reduction strategies for specific enterprises or sites’ will be developed based on more accurate accounting, facilitating the identification of major emission processes and ensuring the precision and feasibility of mitigation measures. More coordinated efforts to reduce GHGs while maintaining industrial supply chains will be vital for achieving comprehensive and green economic and social development in the future.

## Supplementary Material

nwae481_Supplemental_File

## References

[1] Montzka SA, Dlugokencky EJ, Butler JH. Nature 2011; 476: 43–50. 10.1038/nature10322 21814274

[2] Janssens-Maenhout G, Crippa M, Guizzardi D et al. Sci Data 2019; 11: 959–1002. 10.5194/essd-2017-79

[3] Wu J, Chen M, Sun X et al. Sci Rep 2024; 14: 12379. 10.1038/s41598-024-62979-z 38811664 PMC11637025

[4] Liu Y, Cheng Z, Chen AY et al. Fundam Res 2022; 10.1016/j.fmre.2022.08.006.

[5] Zhao X, Jin XK, Guo W et al. Earth's Future 2019; 7: 480–90. 10.1029/2018EF001113

[6] Liang M, Zhou Z, Ren P et al. Natl Sci Rev 2024; 11: nwad285. 10.1093/nsr/nwad285 38487250 PMC10939392

[7] Intergovernmental Panel on Climate Change . Climate Change 2021—The Physical Science Basis: Working Group I Contribution to the Sixth Assesment Report of the Intergovernmental Panel on Climate Change. Cambridge: Cambridge University Press, 2003.

[8] NDRC . The People's Republic of China Third Biennial Update Report on Climate Change (in Chinese). https://www.mee.gov.cn/ywdt/hjywnews/202312/W020231229717236049262.pdf (15 January 2025, date last accessed).

[9] Yang M, Peng M, Wu D et al. Resour Conserv Recycl 2023; 190: 106794. 10.1016/j.resconrec.2022.106794

[10] Duren RM, Thorpe AK, Foster KT et al. Nature 2019; 575: 180–4. 10.1038/s41586-019-1720-3 31695210

