# Supplementary material for: High-resolution mapping of CH4/N2O emissions from industrialization-related anthropogenic sources in China
Source: Natl Sci Rev. 2025 Jan 6;12(4):nwae481. doi: 10.1093/nsr/nwae481 (PMC12089771; doi:10.1093/nsr/nwae481)
Supplement: nwae481_Supplemental_File [file nwae481_supplemental_file.docx]

***Supplementary Information for***

**High-resolution mapping of CH_4_/N_2_O emissions from industrialization-related anthropogenic sources in China**

Ziyang Lou^a,h,i⊥^, Haoyu Zhang^a⊥^, Xu Zhao^c⊥^, Qiang Liu^b⊥^, Minqi Liang^d⊥^, Hui Wang^a⊥^, Huiwen Yang^c^, Bofeng Cai^e^, Jingyi Lu^a^, Ying Cui^j^, Jingyi Wu^h^, Fei Teng^b^, Xiao Lu^d^, Wenping Yuan^f^, Mengyao Liu^g,*^

^a^ Shanghai Engineering Research Center of Solid Waste Treatment and Resource Recovery, School of Environmental Science & Engineering, Shanghai Jiao Tong University, Shanghai, 200240, China

^b^ Institute of Energy, Environment and Economy, Tsinghua University, Beijing, 100084, China

^c^ Institute of Blue and Green Development, Shandong University, Weihai, 264209, China

^d^ School of Atmospheric Sciences, Guangdong Province Data Center of Terrestrial and Marine Ecosystems Carbon Cycle, Sun Yat-sen University, Zhuhai, Guangdong 510245, China

^e^ Center for Climate Change and Environmental Policy, Chinese Academy for Environmental Planning, Beijing 100012, China

^f^ Institute of Carbon Neutrality, Sino-French Institute for Earth System Science, College of Urban and Environmental Sciences, Peking University, Beijing 100091, China

^g^ Satellite Observation Department, Royal Netherlands Meteorological Institute, De Bilt, the Netherlands

^h^ China-UK Low Carbon College, Shanghai Jiao Tong University, Shanghai 200240, China

^i^ China Institute for Urban Governance, Shanghai Jiao Tong University, Shanghai 200240, China

^j^ School of College of Environmental and Chemical Engineering, Shanghai University of Electric Power, Shanghai 200090, China

^⊥^These authors contributed equally to this work.

*Corresponding author. E-mail: mengyao.liu@knmi.nl

# Sectors and categories of CH_4_/N_2_O emissions sources

The scope of industrial-related anthropogenic sources of energy, Industrial Processes and Product Use (IPPU) and waste sector are summarized here.

Methane (CH_4_) emissions in energy sector include the fugitive methane emissions from coal sector and oil and natural gas (ONG) system, and from inadequate combustion of fossil fuel. To be specific, in coal sector, our inventory encompasses fugitive methane from underground coal mines, open-pit mines, abandoned coal mines, and post-mining activities. The recovery and utilization of mine gas are subtracted from our inventories. In ONG system, our inventory includes the fugitive methane emissions from the whole industrial chain, including exploration, extraction, processing, transmission, refinery, import, and distribution. For combustion methane emissions, we estimate coal and oil/natural gas combustion emissions separately and use the overall combustion activity for calculation (more detailed was shown in **Table S1**).

The subsectors in N_2_O emissions from energy including energy consumption and fugitive emissions from fuels. Specifically, 10 sources including electricity generation, heat plants, petroleum refining, manufacture of solid fuels, other energy industries, manufacturing industries and construction, transport, residential, agriculture/forestry/fishing/fish farms, and non-specified were considered in this study (**Table S2**).

N_2_O emissions from the IPPU sector come from the production of chemical industry, mainly from the production of nitric acid and adipic acid (**Table S2**).

The treatment and purification processes of domestic and industrial wastewater are significant sources of CH_4_ and N_2_O emissions. The emissions arise from the direct discharge during the chemical or biological treatment of wastewater and from indirect discharge generated during the operation of treatment facilities (**Table S1-S2**). Municipal solid waste landfills produce a significant amount of CH_4_ under anaerobic conditions. This study encompasses all urban and county-level landfills (both sanitary and simple landfills [[1](#_ENREF_1)]), whose emissions can be considered as the overall CH_4_ emissions from landfills across China (**Table S1**).

# Methods for CH_4_/N_2_O emissions estimation

The detailed estimation methods for each sector are provided in the following sections. The emissions of CH_4_ and N_2_O were converted into CO_2_ equivalents using the global warming potential values of 29.80 CO_2_-eq for CH_4_ from fossil sources (in this study only for energy sector), 27.00 CO_2_-eq for CH_4_ from non-fossil sources (non-energy sector), and 273.00 CO_2_-eq for N_2_O (all sectors) over a 100-year period according to IPCC AR6 [[2](#_ENREF_2)].

## 2.1 CH_4_/N_2_O emissions from energy

### 2.1.1 CH_4_

Combustion methane and fugitive methane in energy sector are estimated separately. For combustion methane emissions, we used the implied averaged emission factor (EF) from the National Greenhouse Gas Inventories (NGHGIs) [[3](#_ENREF_3), [4](#_ENREF_4)] and the activity data of the nation’s overall coal combustion usage and oil/natural gas combustion usage from China’s energy yearbook.

For fugitive methane emissions, different methodologies are applied for different subcategories. In the coal sector, the annual methane emissions from underground coal mines (CMM) are calculated by using mine-specific EF and annual calibrated production data in our self-built dynamic mine-level database (Tier 3) (**Table S1**). Emissions from open-pit mines are also based on yearly calibrated mining activity data of each open-pit mines, but different to underground mines, we adopt the default EF (2m³per ton raw coal) from IPCC inventory guideline for calculation (Tier 1). For post-mining activity, our methodology is same to NGHGIs, with EF of 3 m³ per ton raw coal for high-gas-content mines, 0.94 m³ per ton raw coal for low-gas-content mines, 0.5 m³/t for open-pit mines (Tier 2). For closed underground mines, studies find that their methane emissions would continue decades [[5](#_ENREF_5), [6](#_ENREF_6)]. Here, we adopt the advanced decay-curve methodology for abandoned mine methane emissions estimation (Tier 3). During past years, China has enhanced their actions in mine gas recovery and utilization for safety reasons. These actions also helped in reducing coal methane emissions. We subtracted the utilized amount of mines gas from our inventory, with source data of mine gas utilization from Energy Data series reports [[7](#_ENREF_7)].

In ONG system, all activity data are originated from China’s energy yearbook, but EFs are from different sources. The EF of natural gas exploration and extraction, gas processing, gas transmission are from previous study [[8](#_ENREF_8)], while EF of other sources are from NGHGIs (Tier 2).

Our database of fugitive energy methane emission sites encompasses 15060 coal mines, including both active and abandoned sites, and 547 emission sites related to oil and gas systems. 81% of these sites are closed (but continue emitting CH_4_) and only 19% are active in 2022. Among the active mines, over 44% are large-scale mines with a capacity exceeding 1,200 kt/a. The database of the ONG system includes information on 59 natural gas fields, 55 oil fields, 275 long-distance pipelines, and 21 liquefied natural gas (LNG) terminals in 2022. All these site-level information is collected from publicly available data sources, mainly from the State Administration of Coal Mine Safety (SACMS; the major source of site-level emission factors), the National Energy Administration (NEA; the major source to deduce operational status of each coal mine), Global Energy Monitor (GEM; the major source of newly constructed coal mines and ONG infrastructures), and other literature [[5](#_ENREF_5), [6](#_ENREF_6)]. The activity data of all energy methane sources are calibrated annually to make it consistent with the statistical data at provincial and national level.

### 2.1.2 N_2_O

The N_2_O emissions from the energy sector were obtained from the full-scale annual N_2_O dataset (FAN2020) [[9](#_ENREF_9)]. The FAN2020 dataset, considered 11 N_2_O emission sources from energy sector, following the emission factor method from IPCC 2019 [[10](#_ENREF_10)]. The FAN2020 dataset offered provincial estimates, which were then allocated to grids using the grid distribution obtained from the EDGAR (Emissions Database for Global Atmospheric Research) dataset in this study. The method for allocating to grids is as follows:

| ${{N_{2}O}_{-energy}}_{i,j}={N_{2}O\_Province}_{j}\times\frac{{N_{2}O\_Grid\_EDGAR}_{i,j}}{{N_{2}O\_Province\_EDGAR}_{j}}$ | (1) |
| --- | --- |

where ${{N_{2}O}_{-energy}}_{i,j}$ is N_2_O emission of the *j*^th^ source inside the *i*^th^ grid each year. ${N_{2}O\_Province}_{j}$ is provincial emission of the *j*^th^ source each year. ${N_{2}O\_Grid\_EDGAR}_{i,j}$ and ${N_{2}O\_Province\_EDGAR}_{j}$ represents the emission of the *j*^th^ sub-sector inside the *i*^th^ grid and the total provincial emission of the *j*^th^ source from the EDGAR dataset.

## 2.2 CH_4_/N_2_O emissions from IPPU

### 2.2.1 N_2_O

Similar to energy sector, N_2_O emissions from the IPPU sector were derived from the FAN2020 dataset. We identified the location information for each adipic acid plant based on the plant names mentioned in commercial reports [[11](#_ENREF_11)]. Around 15 adipic acid and the relative nitric acid factories were included in this assessment. Among them, Huafeng Chemical Co., Ltd. is the predominant manufacturer of adipic acid in China, accounting for 32% of the national production capacity. Based on the plant location information, we allocated the provincial emissions to grids according to the production capacity share of each plant as specified in the report. Provincial nitric acid emissions were allocated to the gridded data using the grid distribution obtained from the EDGAR.

## 2.3 CH_4_/N_2_O emissions from waste

### 2.3.1 CH_4_

The calculation method for CH_4_ emissions from sewage treatment plants is as follows:

| ${CH}_{4-wastewater}={EF}_{i}\times TOW$ | (2) |
| --- | --- |
| ${EF}_{i}=B_{0}\times{MCF}_{i}$ | (3) |

where *EF_i_* denotes the emission factor specific to the treatment process, TOW represents the total COD (Chemical Oxygen Demand) removed, $B_{0}$ is the methane generation potential (0.25 kg CH_4_/kg COD) , ${MCF}_{i}$ is the methane correction factor as recommended by the 2019 IPCC guidelines [[10](#_ENREF_10)], with i representing the process type.

For the period 2014-2018, we utilized detailed data from individual wastewater treatment plants (WWTPs) to estimate CH_4_ emissions. Different wastewater treatment processes require different emission factors ${EF}_{i}$. In aerobic process, *EF_1_* = 0.0075 kgCH_4_/kgCOD; In anaerobic process, *EF_2_* = 0.2 kgCH_4_/kgCOD; In constructed wetland, *EF_i_* is determined according to the specific type of constructed wetland. During the remaining years of the study period, we first calculated the provincial-level CH_4_ emissions and subsequently distributed them across treatment facilities according to each plant’s average CH_4_ emission weight within its respective province in 2014-2018.

Our inventory covers data from 4,551 municipal wastewater treatment plants (WWTPs) and 59,114 industrial wastewater discharge enterprises.

Annual emission of methane (CH_4_) from landfills is calculated using the following formula:

| ${CH}_{4-landfill}=MSW\cdot\sum_{i=1}^{4} MCF\cdot f_{i}\cdot{DOC}_{i}\cdot{DOC}_{F}(e^{-\left( T-1 \right)\cdot k_{i}}-e^{-T\cdot k_{i}})\cdot F\cdot\frac{16}{12}\cdot(1-R)(1-OX)$ | (4) |
| --- | --- |

where ${CH}_{4-landfill}$ represents the CH_4_ emissions from landfilled MSW in one year, MSW is the current amount of waste in the landfill, MCF is the methane correction factor, $f_{i}$ is the proportion of different waste components, i represents different types of waste (in this study: food waste, paper, textiles, wood), DOC_i_ is the proportion of degradable organic carbon, DOC_F_ is the fraction of DOC_i_ that decomposes, T is the time the waste has been in the landfill, $k_{i}$ is the rate constant for CH_4_ generation, F is the proportion of CH_4_ in landfill gas, R is the amount of CH_4_ collected, and OX is the oxidation factor for CH_4_.

We localized these factors based on literature review, field surveys, and landfill facility-level data specific to China and landfills were categorized by size (I, Ⅱ, Ⅲ) and by seven major geographical regions. Detailed values can be found in our previous work [[12](#_ENREF_12)].

Facility-level data were primarily sourced from our previous work [[12](#_ENREF_12)], which included urban and county-level landfills (as mentioned in Section 1) and were corrected using the *China Urban-Rural Construction Statistical Yearbook* and the national statistics by Ministry of Ecology and Environment of the PRC*.*

### 2.3.2 N_2_O

The calculation of N_2_O emissions from wastewater followed the method outlined below:

| ${N_{2}O}_{-wastewater}=EF\times N\times\frac{44}{28}$ | (5) |
| --- | --- |

where the EF is emission factor with a value of 0.035 kg N_2_O/kg N, N represents the total nitrogen removed.

For 2014-2018, detailed data from WWTPs were used to calculate 𝑁, determined by plant-specific indicators like influent total nitrogen and treated water volume. For the remaining study years, a methodology similar to that for CH_4_ emissions was applied, deriving provincial 𝑁 by subtracting emitted nitrogen from generated total nitrogen.

# Reliability analysis

## 3.1 Comprehensive Spatial Coverage

The inventory provided an unprecedented level of completeness and detail across multiple sectors.

In the energy sector, our estimation was based on a site-level emission sources database which encompassed 15060 coal mines, 547 facilities in the oil and natural gas system. Although there are a lot of studies which have developed gridded inventory based on site-level information, our study was based on the most complete dataset to the best of our knowledge. For example, although Edgar v7.0 is one of the most widely used spatial inventories, it sampled only 4264 coal mines from [[13](#_ENREF_13)], and was believed to resulted in spatial bias in coal methane emissions [[14](#_ENREF_14)]. Our dataset documented dynamic information of each energy CH_4_ facility, such as the start year, close year, and capacity adjustment. All the dynamic information enabled the tracking of the impact of recent energy policies on both the temporal and spatial emission patterns, which had been overlooked in many previous studies that allocated annual emissions to static spatial distributions.

The gridded products used in the industrial sector were spatially comprehensive. This study identified the locations of 15 adipic acid production plants and applied the EDGAR dataset to distribute emissions from nitric acid production. EDGAR allocates total national emissions from sub-sectors to grid maps using appropriate proxy distributions, drawing from a well-established spatial proxy index library that reasonably reflects the spatial distribution of emissions for each sub-sector.

In the waste sector, most gridded inventories for China relied on proxy factors like population density to estimate landfill emissions distribution, such as EDGAR[[15](#_ENREF_15)], while the waste disposal facilities varied and updated in different regions and periods[[16](#_ENREF_16)]. This spatial mismatch limited the accuracy of existing inventories. The site-specific data in our inventory was collected based on the local government and the Chinese Ministry of Environmental Protection, which covered a much broader scope than the data recorded in official Chinese statistical yearbooks. The inventory's coverage of WWTPs encompassed nearly all county-level administrative districts, and included data on industrial wastewater, which was often overlooked in previous inventories[[17](#_ENREF_17), [18](#_ENREF_18)].

## 3.2 Comparison with NGHGIs

Compared to NGHGIs values, the average CH_4_ emissions from the energy and waste sectors were 0.87 and 0.93 times of those in the NGHGIs, respectively (**Table S4**). N_2_O emissions in the energy, IPPU, and waste sectors were 0.88, 0.92, and 0.77 times those in the NGHGIs (**Table S3**).

Taken the national inventory of 2005 and 2018 for comparation, which have been re-calculated by the NGHGIs team (<https://www.mee.gov.cn>), our results were 0.58-0.70 and 0.80-0.86 times of NGHGIs values, since the variations in emission factors and activity data of each sector were based on site-level sources, better suited for the local situation. The bottom-up methods with point source could benefit for more accurate data for these sectors.

The spatial and temporal emission estimates demonstrate strong agreement with recent top-down studies. For instance, methane emission hotspots in central and southwestern China identified in the energy sector are consistent with findings from[[19](#_ENREF_19)], who also noted emission increases in these regions. Temporal trends align with the top-down study by[[19](#_ENREF_19)], with both analyses identifying 2014 as the peak year for energy methane emissions. This consistency in emission patterns highlights the robustness of our estimates compared to previous studies[[20](#_ENREF_20)].

## 3.3 Localized EFs and Activity Data

The estimates were improved by utilizing highly localized, site-specific data. In the energy sector, which represents the largest share of emissions, previous inventories used national-level activity data from the IEA to estimate N_2_O emissions. This study, employed provincial energy balance tables from the China Energy Statistical Yearbook, offering a more accurate representation of China's energy consumption. For CH_4_ emissions, underground coal mine methane (CMM) was calculated using mine-specific emission factors and annually updated production data from a dynamic mine-level database, with an advanced decay-curve methodology (Tier 3) applied to estimate emissions from abandoned coal mines.

Industrial emissions were also more reliably captured. Investigations revealed that only two adipic acid plants implemented abatement technologies between 2008 and 2012 under the Clean Development Mechanism project. Although emissions were reduced at these two plants, overall emissions continued to rise rapidly due to the significant increase in adipic acid production during this period. After 2013, emissions grew at a high rate following the cessation of abatement efforts, a trend that is not mirrored in past inventories such as EDGAR and GAINS, which either depicted a sharp decline or merely a gradual increase in emissions after 2008.

In the waste sector, existing datasets often relied on IPCC-recommended default emission factors[[15](#_ENREF_15)]. While the local conditions (such as waste composition and landfill operations) are quite different from those in Europe and the U.S.[[21](#_ENREF_21)]. Waste composition data in this study, derived from long-term on-site investigations, enabled the use of more region-specific parameters, thereby enhancing the accuracy of emissions estimates. The dataset also included additional information, such as construction dates, annual waste volumes, and designed capacities, allowing landfills to be categorized into three capacity-based groups with specific emission factors applied to each.

# Spatial and temporal characteristics

## 4.1 Spatial characteristics

The spatial distribution of emissions in the energy sector exhibited significant clustering (**Fig. S3**). For CH_4_ emissions, the main contributors were fugitive emissions from coal mining, closely associated with the distribution of coal resources. For example, in 2022, emissions from Shanxi and Guizhou were several times higher than those of other provinces, with fugitive emissions of 7.93 Tg CH_4_ yr^-1^ and 3.31 Tg CH_4_ yr^-1^, respectively. Additionally, Inner Mongolia, Shaanxi, Xinjiang, and Sichuan also had substantial emissions. With the phasing out of outdated coal production capacities, provinces like Liaoning, Henan, Hunan, Chongqing, Sichuan, and Yunnan saw a decrease in the number of small-scale, outdated coal mines over time, resulting in a corresponding decrease in emissions. Conversely, the shift of coal production centers to Shanxi, Shaanxi, and Xinjiang had led to an increase in coal production and energy-related methane emissions over time.

For energy-related N_2_O emissions, the distribution was strongly correlated with the use of fossil fuels, primarily from power generation. These emissions were concentrated in eastern regions such as Inner Mongolia, Shandong, Jiangsu, and Guangdong, with emission levels ranging from 0.02 to 0.04 Tg N_2_O yr^-1^.

Among these two gases, industrial greenhouse gas emissions primarily originated from N_2_O produced during adipic acid production, mainly concentrated in Chongqing and Shandong. The emissions in these two provinces were 0.16 Tg N_2_O yr^-1^ and 0.12 Tg N_2_O yr^-1^, respectively, which coincided with the locations of leading enterprises. Leading companies included Chongqing Huafeng, Shandong Hualu Hengsheng, Shandong Hongye Chemical, and Shandong Haili Chemical Co., Ltd.

CH_4_ emissions from the waste sector were mainly concentrated in three high-density regions in Eastern China: Beijing-Tianjin, the Pearl River Delta, and the Yangtze River Delta. In the central region, the increase in industrial wastewater treatment had led to a significant rise in CH_4_ emissions from WWTPs.

Due to high population density and high sewage collection rates, N_2_O emissions from WWTPs in East China were significantly higher than in other regions, generating approximately 0.08 Tg N_2_O yr^-1^ in 2022. The northeastern region was one of China's prominent heavy industrial centers. Despite having a lower population density, it had high N_2_O emissions from industrial WWTPs due to its status as a major heavy industrial base.

## 4.2 Temporal characteristics

CH_4_ emissions exhibited a direct increase until 2014, followed by a subsequent decrease in the energy system (**Fig. S4**). The rise in CH_4_ levels was mainly due to the increase in energy consumption during rapid economic development. Subsequently, most of small-scale and unsafe coal mining sites were forced to be closed with an escalation in domestic mining activities for overcapacity reduction policy, while the fugitive CH_4_ were still released from the abandoned mining sites in the last two years. The remaining coal production shifted to large-scale coal mining areas, located in the northeast and northwest regions, equipped with the high-quality coal mining system and low GHGs emission [[6](#_ENREF_6)].

CH_4_ and N_2_O emissions from waste sectors showed an overall fluctuating growth trend. Emissions from landfills peaked at 4.14 Tg CH_4_ yr^-1^ in 2019, as aged refuse continued to contribute to emissions for several decades after disposal. With the implementation of “The Action Plan for Prevention and Treatment of Water Pollution” in 2015, WWTPs were upgraded to meet a high-quality discharge standards [[22](#_ENREF_22)], which resulted in a significant increase in the occupancy rate, with around 48-91% of total CH_4_ emissions. The average annual N_2_O emissions in the WWTPs increased 4.2 times more from 2000 to 2022, while the GDP growth level was more than 10 times, meaning that there are still some gaps in wastewater treatment capacity [[23](#_ENREF_23)].

# Figures and tables


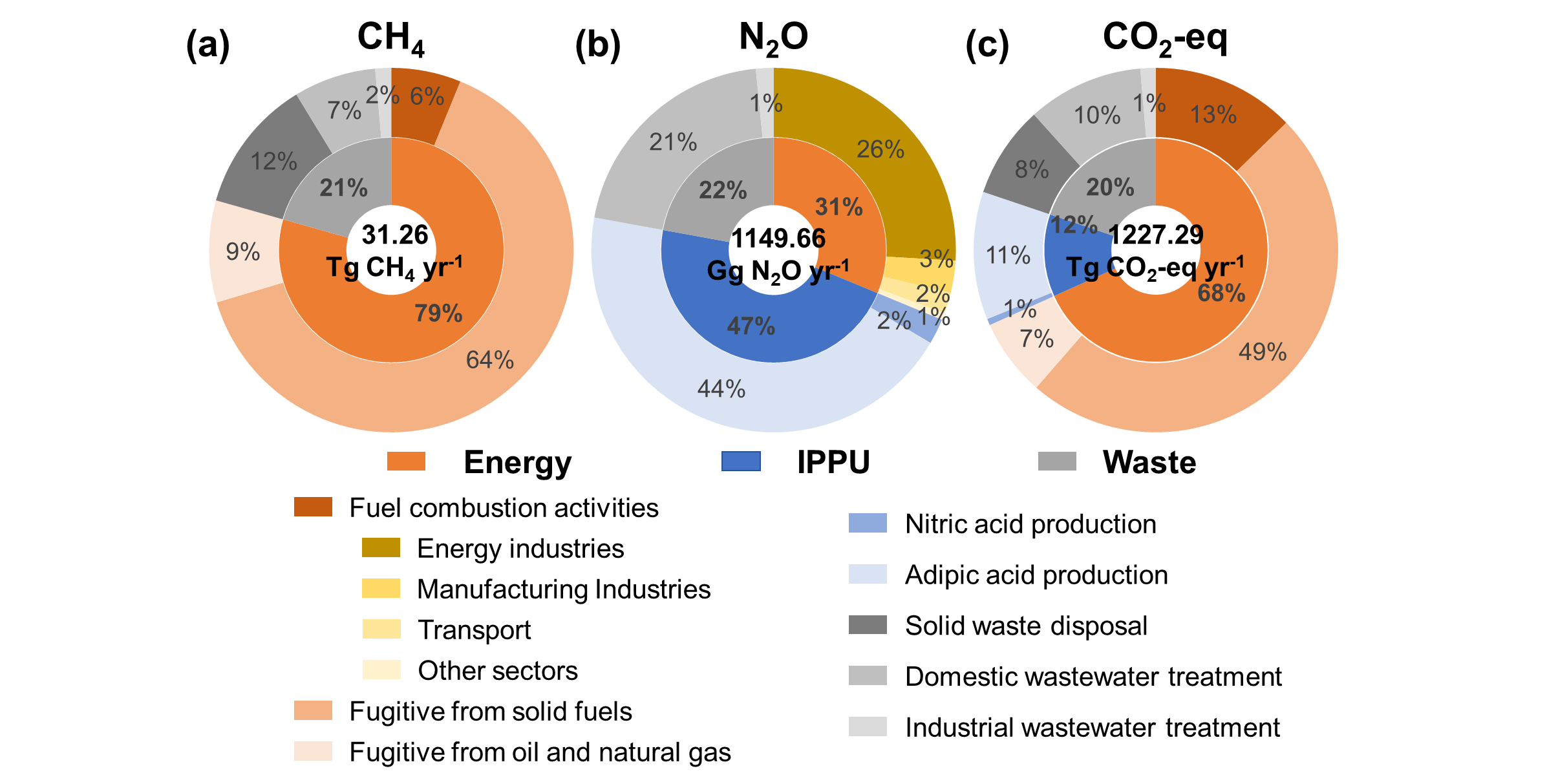


**Figure S1.** The proportion of CH_4_ (a) and N_2_O (b) and their total CO_2_-eq (c) emissions from different sectors include energy, Industrial Processes and Product Use (IPPU), and waste.


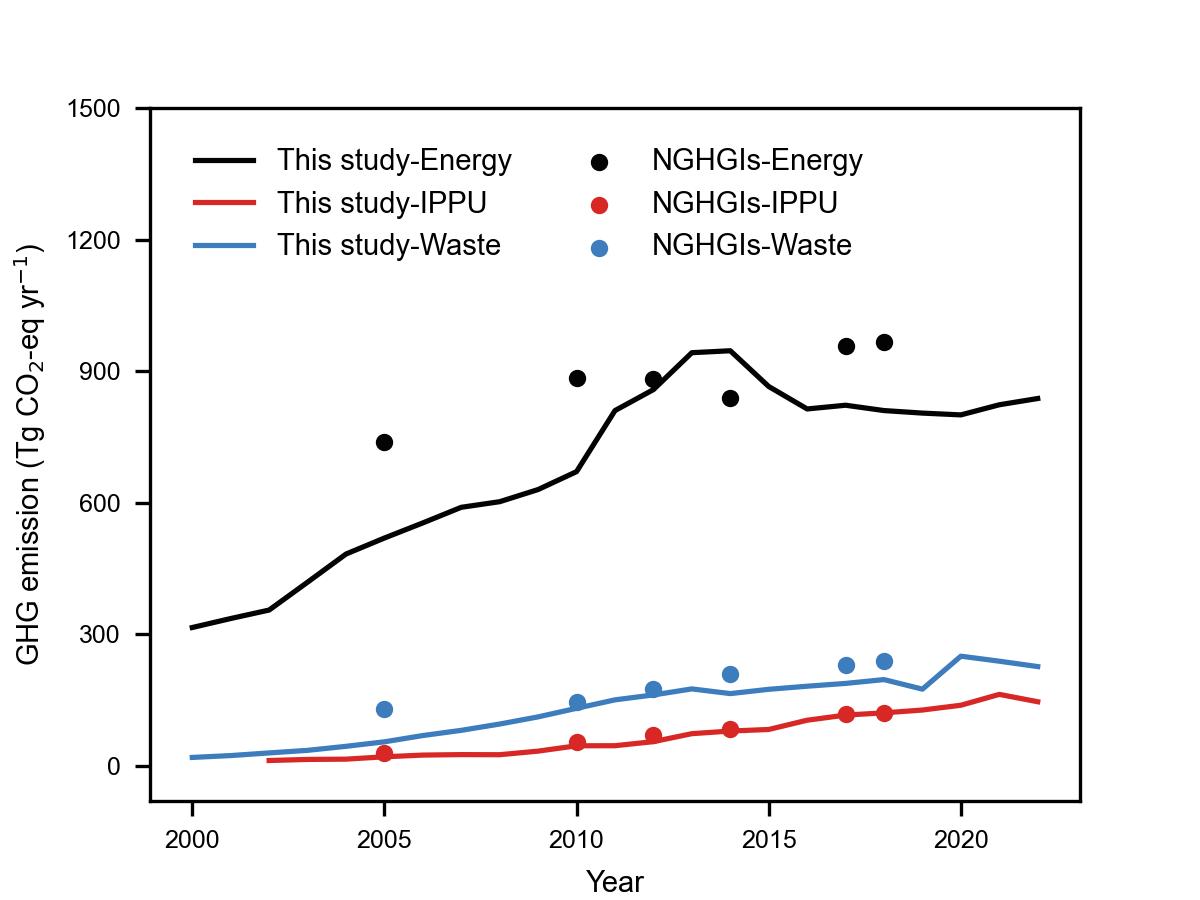


**Figure S2.** Total CH_4_ and N_2_O emissions (CO_2_-eq) during 2000-2022. Spots are data reported in the National Greenhouse Gas Inventories (NGHGIs) and lines are our inventory. To ensure comparability with NGHGIs, industrial wastewater emissions are not shown in the figure; detailed values can be found in **Table S3-S4**.


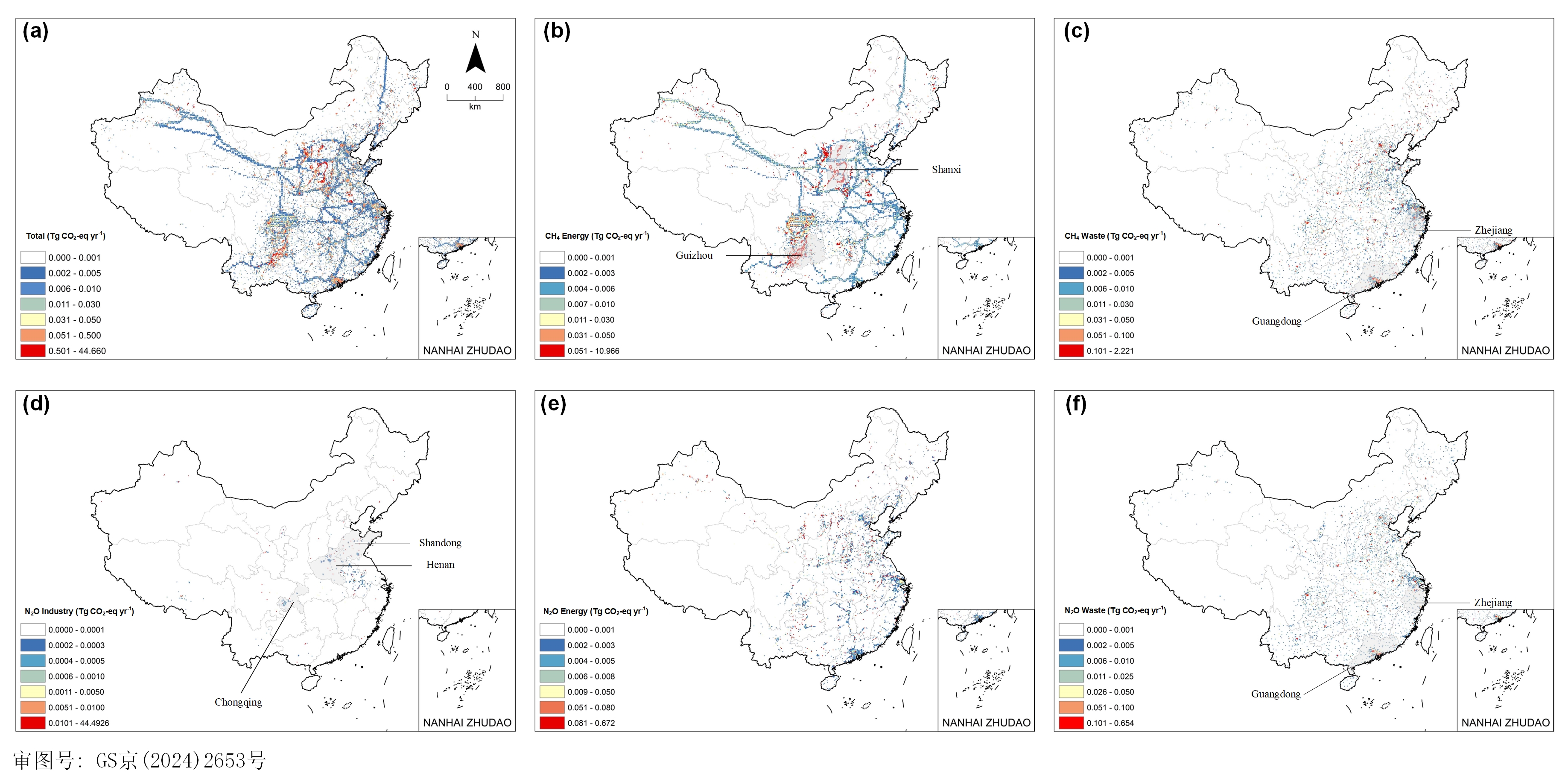


**Figure S3.** Geographical distribution on CH_4_ and N_2_O emissions by sector in 2022. There is no data available in Hong Kong, Macao and Taiwan.


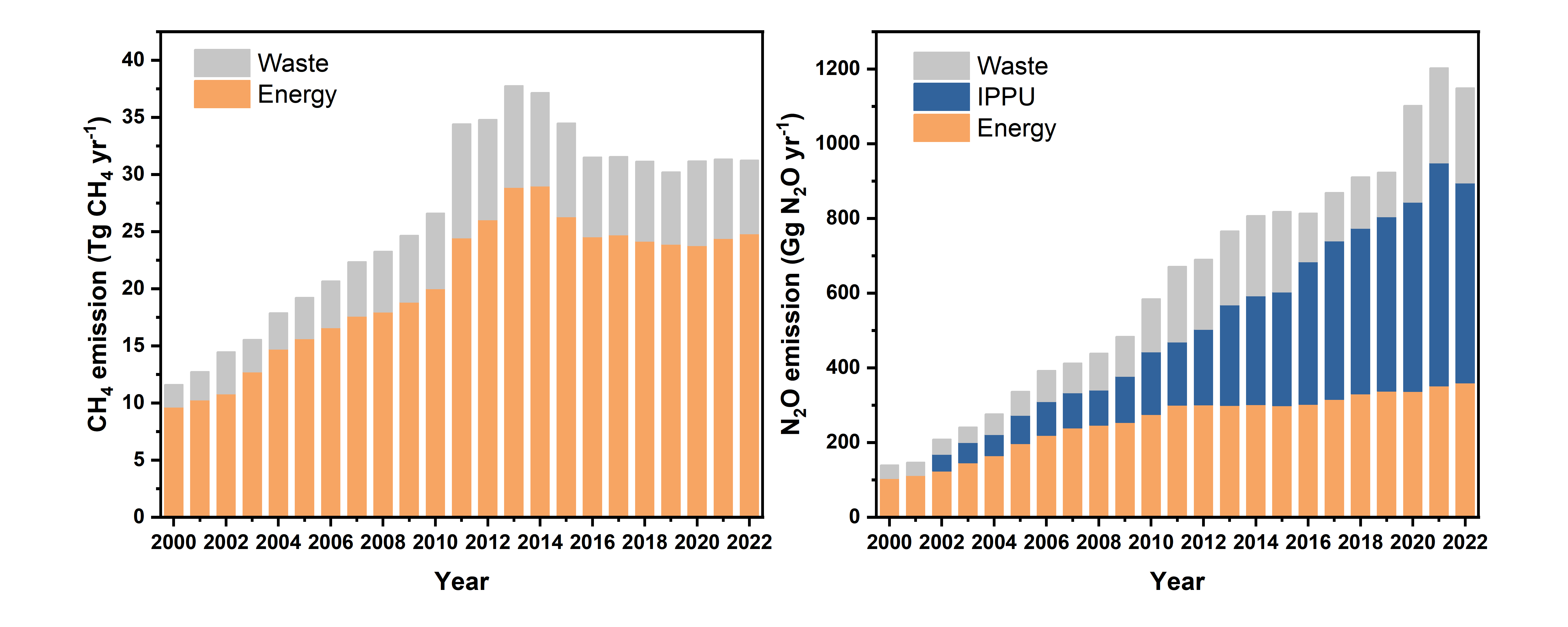


**Figure S4.** CH_4_ and N_2_O emissions during 2000-2022.

**Table S1.** Sectors and categories of CH_4_ emissions included in the dataset.

| **Sector** | **Source** | **Method^1^** | **IPCC codes^2^** | **Accounting period** |
| --- | --- | --- | --- | --- |
| Energy | Fuel combustion activities* | EF (T1) | 1A | 2011-2022 |
|  | Fugitive emissions from fuels* |  | 1B | 2000-2022 |
|  | Coal sector | MP | 1B1 |  |
|  | Oil and natural gas | EF (T2) | 1B2 |  |
| Waste | Solid Waste Disposal* |  | 4A | 2000-2022 |
|  | Managed Waste Disposal Sites | EF (T2) | 4A1 |  |
|  | Unmanaged Waste Disposal Sites |  | 4A2 |  |
|  | Wastewater Treatment and Discharge* |  | 4D | 2000-2022 |
|  | Domestic Wastewater Treatment  and Discharge | EF (T1) | 4D1 |  |
|  | Industrial Wastewater Treatment  and Discharge |  | 4D2 |  |

^1^Method abbreviation: EF (emission factor method), T1/2 (Tier 1/2), MP (mine-specific EFs). ^2^IPCC code means the corresponding code of category in the 2019 Refinement to the 2006 IPCC Guidelines on National Greenhouse Gas Inventories. *The emissions of these categories were not estimated directly, but the sum of their sub-categories.

**Table S2.** Sectors and categories of N_2_O emissions included in the dataset.

| **Sector** | **Source** | **Method^1^** | **IPCC codes^2^** | **Accounting period** |
| --- | --- | --- | --- | --- |
| Energy | Fuel combustion activities* |  | 1A | 2000-2022 |
|  | Energy industries* |  | 1A1 |  |
|  | Main activity electricity and heat production* |  | 1A1a |  |
|  | Electricity Generation | EF (T1) | 1A1ai |  |
|  | Heat Plants |  | 1A2aiii |  |
|  | Petroleum Refining | EF (T1) | 1A1b |  |
|  | Manufacture of solid fuels and other energy industries* |  | 1A1c |  |
|  | Manufacture of Solid Fuels |  | 1A1ci |  |
|  | Other energy industries |  | 1A1cii |  |
|  | Manufacturing Industries & Construction | EF (T1) | 1A2 |  |
|  | Transport | EF (T2) | 1A3 |  |
|  | Other sectors* | EF (T1) | 1A4 |  |
|  | Residential |  | 1A4b |  |
|  | Agriculture/Forestry/Fishing/Fish Farms |  | 1A4c |  |
|  | Non-Specified |  | 1A5 |  |
|  | Fugitive emissions from fuels* | EF (T1) | 1B |  |
|  | Oil and natural gas |  | 1B2 |  |
| Industrial processes and product use | Chemical industry* |  | 2B | 2002-2022 |
|  | Nitric acid production | EF (T2) | 2B2 |  |
|  | Adipic acid production |  | 2B3 |  |
| Waste  Waste | Solid Waste Disposal* |  | 4A | 2000-2022 |
|  | Managed Waste Disposal Sites | EF (T2) | 4A1 |  |
|  | Unmanaged Waste Disposal Sites |  | 4A2 |  |
|  | Wastewater Treatment and Discharge* |  | 4D |  |
|  | Domestic Wastewater Treatment  and Discharge | EF (T1) | 4D1 |  |
|  | Industrial Wastewater Treatment  and Discharge |  | 4D2 |  |

^1^Method abbreviation: EF (emission factor method), T1/2 (Tier 1/2), MP (mine-specific EFs). ^2^IPCC code means the corresponding code of category in the 2019 Refinement to the 2006 IPCC Guidelines on National Greenhouse Gas Inventories. *The emissions of these categories were not estimated directly, but the sum of their sub-categories.

**Table S3.** Comparison of N_2_O emissions in this study with NGHGIs.

| **Sectors** | **Sources in this study** | **N_2_O emissions in this study (Gg N_2_O yr^-1^)** | | | | | | **Sources in NGHGIs** | **N_2_O emissions in NGHGIs (Gg N_2_O yr^-1^)** | | | | | | |  |
| --- | --- | --- | --- | --- | --- | --- | --- | --- | --- | --- | --- | --- | --- | --- | --- | --- |
|  |  | **2005** | **2010** | **2012** | **2014** | **2017** | **2018** |  | | **2005^a^** | **2010** | **2012** | **2014** | **2017** | **2018** | |
| Energy | Electricity generation | 136.14 | 203.16 | 223.24 | 229.11 | 244.96 | 265.09 | Energy industries | | 264.52 | 165.00 | 89.00 | 223.00 | 263.00 | 291.00 | |
|  | Heat plants |  |  |  |  |  |  |  |  |  |  |  |  |  |  |  |
|  | Petroleum refining |  |  |  |  |  |  |  |  |  |  |  |  |  |  |  |
|  | Manufacture of solid fuels |  |  |  |  |  |  |  |  |  |  |  |  |  |  |  |
|  | Other energy industries |  |  |  |  |  |  |  |  |  |  |  |  |  |  |  |
|  | Manufacturing industries  and construction | 41.29 | 45.49 | 48.16 | 43.89 | 39.35 | 35.71 | Manufacturing Industries | |  | 53.00 | 52.00 | 65.00 | 71.00 | 75.00 | |
|  | Transport | 11.40 | 17.10 | 20.30 | 20.35 | 22.37 | 21.91 | Transport | |  | 20.00 | 22.00 | 21.00 | 21.00 | 23.00 | |
|  | Residential | 6.83 | 6.96 | 7.72 | 6.91 | 7.45 | 6.73 | Other sectors | |  | 7.00 | 7.00 | 7.00 | 22.00 | 22.00 | |
|  | Agriculture/forestry/fishing/fish farms |  |  |  |  |  |  |  |  |  |  |  |  |  |  |  |
|  | Non-specified | 0.93 | 2.00 | 0.81 | 0.87 | 0.87 | 0.86 | Others | |  | 63.00 | 55.00 | 51.00 | ˗ | ˗ | |
|  | Fugitive emissions from fuels | 0.11 | 0.10 | 0.12 | 0.06 | 0.02 | 0.01 |  | |  |  |  |  |  |  | |
|  | **Subtotal** | **196.70** | **274.81** | **300.36** | **301.19** | **315.02** | **330.32** | **Subtotal** | | **264.52** | **308.00** | **224.00** | **367.00** | **377.00** | **411.00** | |
| Industry | Nitric acid production | 76.13 | 168.09 | 202.72 | 291.19 | 424.36 | 443.14 | Chemical industry | | 109.68 | 200.00 | 255.00 | 311.00 | 435.00 | 441.00 | |
|  | Adipic acid production |  |  |  |  |  |  |  |  |  |  |  |  |  |  |  |
|  | **Subtotal** | **76.13** | **168.09** | **202.72** | **291.19** | **424.36** | **443.14** |  | | **109.68** | **200.00** | **255.00** | **311.00** | **435.00** | **441.00** | |
| Waste | Domestic Wastewater Treatment  and Discharge | 17.19 | 50.64 | 57.39 | 83.88 | 100.51 | 110.23 | Wastewater Treatment and Discharge | | 93.55 | 96.00 | 97.00 | 110.00 | 113.00 | 113.00 | |
|  | Industrial Wastewater Treatment  and Discharge | 46.53 | 90.70 | 130.10 | 130.98 | 29.58 | 27.67 |  |  |  |  |  |  |  |  |  |
|  | **Subtotal** | **63.72** | **141.34** | **187.49** | **214.86** | **130.09** | **137.90** | **Subtotal** | | **93.55** | **96.00** | **97.00** | **110.00** | **113.00** | **113.00** | |
| **Total** | **Total** | **336.55** | **584.24** | **690.57** | **807.24** | **869.47** | **911.36** | **Total** | | **467.74** | **604.00** | **576.00** | **788.00** | **925.00** | **965.00** | |

^a^ N_2_O emissions in 2005 from the NGHGIs are provided from the retrospective calculation in the People’s Republic of China Third Biennial Update Report on Climate Change.

**Table S4.** Comparison of CH_4_ emissions in this study with NGHGIs.

| **Sectors** | **Sources in this study** | **CH_4_ emissions in this study (Tg CH_4_ yr^-1^)** | | | | | | **Sources in NGHGIs** | **CH_4_ emissions in NGHGIs (Tg CH_4_ yr^-1^)** | | | | | |
| --- | --- | --- | --- | --- | --- | --- | --- | --- | --- | --- | --- | --- | --- | --- |
|  |  | **2005** | **2010** | **2012** | **2014** | **2017** | **2018** |  | **2005^a^** | **2010** | **2012** | **2014** | **2017** | **2018** |
| Energy | Electricity generation | - | - | 0.36 | 0.45 | 1.46 | 0.85 | Energy industries | 23.67 | 0.04 | 0.05 | 0.05 | 0.09 | 0.10 |
|  | Heat plants |  |  |  |  |  |  |  |  |  |  |  |  |  |
|  | Petroleum refining |  |  |  |  |  |  |  |  |  |  |  |  |  |
|  | Manufacture of solid fuels |  |  |  |  |  |  |  |  |  |  |  |  |  |
|  | Other energy industries |  |  |  |  |  |  |  |  |  |  |  |  |  |
|  | Manufacturing industries  and construction |  |  |  |  |  |  | Manufacturing Industries |  | 0.28 | 0.20 | 0.32 | 0.30 | 0.29 |
|  | Transport |  |  |  |  |  |  | Transport |  | 0.07 | 0.08 | 0.08 | 0.13 | 0.11 |
|  | Residential |  |  |  |  |  |  | Other sectors |  | 2.6 | 2.29 | 2.16 | 1.25 | 1.13 |
|  | Agriculture/forestry/fishing/fish farms |  |  |  |  |  |  |  |  |  |  |  |  |  |
|  | Non-specified |  |  |  |  |  |  |  |  |  |  |  |  |  |
|  | Fugitive emissions from solid fuel | 15.09 | 19.06 | 23.33 | 25.87 | 21.08 | 19.99 | Solid fuels |  | 22.87 | 23.85 | 21.02 | 25.19 | 25.12 |
|  | Fugitive emissions from oil and natural gas system | 0.54 | 0.94 | 1.40 | 1.77 | 2.23 | 2.45 | Oil and natural gas system |  | 0.96 | 1.12 | 1.13 | 1.71 | 1.88 |
|  | **Subtotal** | **15.63** | **20.00** | **26.08** | **29.06** | **24.77** | **24.22** | **Subtotal** | **23.67** | **26.83** | **27.59** | **24.76** | **28.68** | **28.66** |
| Waste | Solid Waste Disposal | 0.92 | 2.08 | 2.81 | 3.20 | 3.87 | 4.06 | Solid Waste Disposal | 3.86 | 2.21 | 2.53 | 3.84 | 4.43 | 4.69 |
|  | Domestic Wastewater Treatment  and Discharge | 1.00 | 2.37 | 2.71 | 2.17 | 2.18 | 2.22 | Wastewater Treatment and Discharge |  | 2.19 | 2.89 | 2.72 | 2.91 | 2.91 |
|  | Industrial Wastewater Treatment  and Discharge | 1.74 | 2.26 | 3.35 | 2.91 | 0.91 | 0.83 | Others | - | - | - | - | - | - |
|  | **Subtotal** | **3.66** | **6.71** | **8.87** | **8.28** | **6.96** | **7.11** | **Subtotal** | **3.86** | **4.40** | **5.42** | **6.56** | **7.34** | **7.59** |
| **Total** | **Total** | **19.29** | **26.71** | **34.95** | **37.34** | **31.73** | **31.33** | **Total** | **27.53** | **31.23** | **33.01** | **31.32** | **36.02** | **36.25** |

^a^ CH_4_ emissions in 2005 from the NGHGIs are provided from the retrospective calculation in the Third National Communications on Climate Change.

# References

1. Wang A, Zhang L, Shi Y *et al.* Rural solid waste management in China: Status, problems and challenges. *Sustainability*. 2017; **9**(4): 506.

2. Intergovernmental Panel on Climate C. *Climate Change 2021 – The Physical Science Basis: Working Group I Contribution to the Sixth Assessment Report of the Intergovernmental Panel on Climate Change*. Cambridge: Cambridge University Press, 2023.

3. NDRC. The People’s Republic of China Second Biennial Update Report on Climate Change. 2018.

4. Commission CCDoNDR. The People’s Republic of China first biennial update report on climate change. 2017.

5. Kholod N, Evans M, Pilcher RC *et al.* Global methane emissions from coal mining to continue growing even with declining coal production. *Journal of Cleaner Production*. 2020; **256**: 120489.

6. Liu Q, Teng F, Nielsen CP *et al.* Large methane mitigation potential through prioritized closure of gas-rich coal mines. *Nature Climate Change*. 2024: 1-7.

7. Wang Q. *2021 Energy Data*. The Institute for Global Decarbonization Progress; 2022 (DHHS publication no.: Report Number)| (GPO o. Document Number)|.

8. Gan Y, El-Houjeiri HM, Badahdah A *et al.* Carbon footprint of global natural gas supplies to China. *Nat Commun*. 2020; **11**(1): 1-9.

9. Liang M, Zhou Z, Ren P *et al.* Four decades of full-scale nitrous oxide emission inventory in China. *National Science Review*. 2024; **11**(3): nwad285.

10. Change IPoC. 2019 Refinement to the 2006 IPCC guidelines for national greenhouse gas inventories. IPCC Geneva, Switzerland:; 2019. 824.

11. Huaon. *Report of Market Development Monitoring and Investment Strategy Planning Research on China adipic acid Industry. (In Chinese).* 2023 (DHHS publication no.: Report Number)| (GPO o. Document Number)|.

12. Cai B, Lou Z, Wang J *et al.* CH_4_ mitigation potentials from China landfills and related environmental co-benefits. *Science Advances*. 2018; **4**(7): eaar8400.

13. Liu Z, Guan D, Wei W *et al.* Reduced carbon emission estimates from fossil fuel combustion and cement production in China. *Nature*. 2015; **524**(7565): 335-338. doi: 10.1038/nature14677

14. Sheng J, Song S, Zhang Y *et al.* Bottom-Up Estimates of Coal Mine Methane Emissions in China: A Gridded Inventory, Emission Factors, and Trends. *Environmental Science & Technology Letters*. 2019; **6**(8): 473-478. doi: 10.1021/acs.estlett.9b00294

15. Janssens-Maenhout G, Crippa M, Guizzardi D *et al.* EDGAR v4.3.2 Global Atlas of the three major greenhouse gas emissions for the period 1970–2012. *Earth Syst Sci Data*. 2019; **11**(3): 959-1002. doi: 10.5194/essd-11-959-2019

16. Lou Z, Cai B-F, Zhu N *et al.* Greenhouse gas emission inventories from waste sector in China during 1949–2013 and its mitigation potential. *Journal of Cleaner Production*. 2017; **157**: 118-124. doi: <https://doi.org/10.1016/j.jclepro.2017.04.135>

17. Du W-J, Lu J-Y, Hu Y-R *et al.* Spatiotemporal pattern of greenhouse gas emissions in China’s wastewater sector and pathways towards carbon neutrality. *Nature Water*. 2023; **1**(2): 166-175. doi: 10.1038/s44221-022-00021-0

18. Wang D, Ye W, Wu G *et al.* Greenhouse gas emissions from municipal wastewater treatment facilities in China from 2006 to 2019. *Scientific Data*. 2022; **9**(1): 317. doi: 10.1038/s41597-022-01439-7

19. Zhang Y, Fang S, Chen J *et al.* Observed changes in China’s methane emissions linked to policy drivers. *Proceedings of the National Academy of Sciences*. 2022; **119**(41): e2202742119. doi: doi:10.1073/pnas.2202742119

20. Liu Q, Teng F, Nielsen CP *et al.* Large methane mitigation potential through prioritized closure of gas-rich coal mines. *Nature Climate Change*. 2024; **14**(6): 652-658. doi: 10.1038/s41558-024-02004-3

21. Liu Y, Cheng Z, Chen AY *et al.* Big disparities in CH4 emission patterns from landfills between the United States and China and their behind driving forces. *Fundamental Research*. 2022. doi: <https://doi.org/10.1016/j.fmre.2022.08.006>

22. Han D, Currell MJ, Cao G. Deep challenges for China's war on water pollution. *Environ Pollut*. 2016; **218**: 1222-1233.

23. Liu C, Cai W, Zhai M *et al.* Decoupling of wastewater eco-environmental damage and China's economic development. *Sci Total Environ*. 2021; **789**: 147980.
